# Audit of Operation Notes During Wartime at Abuhamad Hospital, Sudan

**DOI:** 10.7759/cureus.91197

**Published:** 2025-08-28

**Authors:** Muhammad Omer, Mohammed Elfatih Ahmed Yousif, Almthani A Hamza, Rashid Albalal Ahmed, Altayeb Mohammed, Shima Mohamed Abdallah Alradi, Nidal Youseef AlTaher Aboh, Zeinab Abdelfattah Ahmed Babiker

**Affiliations:** 1 College of Medicine and Surgery, Karary University, Omdurman, SDN; 2 Department of Internal Medicine, Knowledge Translation Section, Altababa Advanced Training Center, Khartoum, SDN; 3 Department of Pathology, Elsheikh Abdallah Elbadri University, Atbara, SDN; 4 Department of Surgery, College of Medicine and Surgery, University of Bahri, Bahri, SDN; 5 Department of Surgery, Faculty of Medicine, University of Khartoum, Khartoum, SDN; 6 Department of Surgery, Ahfad University for Women, Khartoum, SDN; 7 Department of Surgery, University of Gezira, Wad Madani, SDN; 8 Department of Surgery, Faculty of Medicine and Health Sciences, Nile Valley University, Khartoum, SDN

**Keywords:** operative notes, quality improvement, sudan, surgical documentation, wartime

## Abstract

Background

Operative notes are vital for surgical practice, serving as critical records for postoperative care, clinical audits, and legal documentation. The Royal College of Surgeons of England (RCSEng) emphasizes the importance of detailed and structured notes. However, in conflict-affected and low-resource settings like Sudan, the quality of documentation is often poor due to staff shortages, inconsistent training, and the absence of standardized templates. This audit at Abuhamad Hospital aimed to assess operative note quality during wartime, implement corrective measures, and evaluate the outcomes.

Material and methods

The audit was conducted from August to November 2024, amid internal conflict, using a closed-loop, two-cycle model. In the first cycle, 28 retrospective notes were reviewed using 19 RCSEng-based criteria (e.g., patient details, surgeon name, procedure type, findings, complications, and postoperative instructions). Key deficiencies were identified, and a corrective intervention was introduced, including a standardized printed operative note template, focused staff training, and visual reminders in operating theatres. The second cycle prospectively assessed 33 operative notes post-intervention. Data were analyzed using Excel and SPSS version 25, and ethical approval was obtained.

Result

In total, 61 notes were audited. The first cycle revealed major gaps: only 9 (32.1%) documented the surgeon, anesthetist, or assistant, and just 1 (3.5%) recorded tissue/prosthesis details or antibiotic prophylaxis. Closure techniques and surgery type appeared in 4 (14.2%), while deep vein thrombosis (DVT) prophylaxis was entirely absent. Only 5 (17.8%) of notes were signed. Post-intervention, notable improvements were observed: documentation of closure techniques rose to 25 (75.8%), surgeon’s name, diagnosis, and postoperative instructions to 25 (75.8%), anesthetist and operation time to 22 (66.7%), and date of operation to 24 (72.7%). Incision details and operative findings also improved substantially. Documentation of DVT prophylaxis increased to 8 (24.2%), and signatures to 13 (39.4%). The mean compliance across all parameters rose from 24.1% to 52.3%, a 28.2% improvement.

Conclusion

Structured documentation tools and focused training significantly enhanced operative note quality at Abuhamad Hospital, even during wartime. The findings align with both local and international evidence supporting low-cost, high-impact quality improvement methods. Despite a slight decline in documentation of postoperative instructions, the audit highlights the importance of standardization, ongoing training, and regular audits, particularly in resource-limited and unstable settings.

## Introduction

Operative notes are a critical component of surgical practice, serving as comprehensive records of intraoperative events and decision-making. They are indispensable for postoperative care, clinical audits, medico-legal documentation, and continuity of patient management. The Royal College of Surgeons of England (RCSEng) has issued guidelines emphasizing the importance of detailed and structured operative notes to ensure high standards of surgical documentation [1].

In low-resource and conflict-affected settings like Sudan, challenges such as staff shortages, inconsistent training, and lack of standardized templates often compromise the quality of surgical records. Previous audits conducted across Sudan, including at Dongola, Port Sudan, Elobeid, Wad Madani, and Doka Teaching Hospitals, demonstrated that introducing proformas and training sessions significantly improved documentation completeness, with gains ranging from 20-30% [2-5].

International audits echo these findings. A Pakistan-based study demonstrated improved documentation by 35.7% post-intervention using a revised proforma and clinician training [6]. Likewise, in Malawi, standardized templates and focused teaching improved adherence to surgical documentation guidelines, especially for medico-legal elements [7].

Given the context of ongoing conflict and displacement, none have examined this within an active wartime context, where instability and staff turnover present additional challenges to sustainability. The current audit at Abuhamad Hospital aimed to assess the quality of operative notes, implement corrective interventions, and re-audit to evaluate progress. The findings are expected to support quality assurance and patient safety initiatives in similar settings.

## Materials and methods

Study setting and duration

This audit was conducted at Abuhamad Hospital, located in Northern Sudan, during a four-month period from August to November 2024. The study took place amid active internal conflict, which presented logistical and operational challenges. Despite these constraints, the audit was successfully executed using a two-cycle closed-loop model designed to assess and improve the quality of surgical documentation in operative notes.

Audit design

The audit employed the RCSEng guidelines [1] as the benchmark for evaluating documentation standards. In the first cycle, 28 operative notes were retrospectively reviewed from approximately 95 surgeries performed during the same period. Selection was consecutive to minimize bias and included both emergency and elective cases across general surgery, obstetrics and gynaecology, and orthopaedics. A structured checklist comprising 19 documentation parameters was used, based on RCSEng recommendations. These parameters included essential elements such as patient identifiers, diagnosis, time and date of procedure, names of the surgeon, anesthetist, and assistants, procedure type, incision details, intraoperative findings, complications, closure method, use of tissue or prosthesis, antibiotic and deep vein thrombosis (DVT) prophylaxis, and postoperative instructions.

Intervention

Following the identification of documentation deficiencies in the first cycle, a structured intervention was implemented. This included the introduction of a printed operative note template designed to align with RCSEng standards, aimed at standardizing documentation practices. A focused training session was conducted for surgical and house officers to reinforce the principles of comprehensive operative note writing. The training session lasted approximately one hour and was delivered by the surgical audit lead. Content included a short lecture on RCSEng standards, distribution of sample operative notes, and a step-by-step walkthrough of the new printed template. To further support compliance, visual reminders outlining key documentation requirements were displayed prominently within the operating theatre.

Second cycle

The second cycle of the audit was conducted prospectively and involved the review of 33 operative notes following the intervention. The same 19-item checklist was used to assess documentation quality, enabling direct comparison with the pre-intervention data.

Data collection and management

Data from both audit cycles were manually extracted and entered into Microsoft Excel 2016. Each operative note was evaluated against the checklist, and compliance was recorded for each parameter. All data were anonymized prior to analysis to ensure patient confidentiality.

Statistical analysis

Statistical analysis was performed using SPSS version 25. Compliance rates for each documentation criterion were calculated as percentages. Comparative analysis was conducted to evaluate changes between the first and second cycles, and percentage improvement for each parameter was determined. Given the audit’s quality improvement focus and limited sample size, only descriptive statistics, frequencies and percentages, were used. Results were presented in tabular format to illustrate documentation trends and improvements.

Ethical considerations

Ethical approval for this audit was obtained from the Institutional Review Board of Abuhamad Teaching Hospital (IRB Approval No.: 1). All personal and clinical data were fully anonymized prior to analysis to ensure the protection of patient privacy and confidentiality.

## Results

A total of 61 operative notes were reviewed over two audit cycles. In the first cycle, documentation was largely completed by medical officers and house officers. While postoperative instructions were present in 26 (92.8%) notes, major deficits were noted in several parameters. Only 9 (32.1%) notes documented the date, operating surgeon, anesthetist, and assistant’s name. Details on tissue/prosthesis used, antibiotic prophylaxis, and anticipated blood loss were extremely scarce, appearing in only 1 (3.5%). Closure techniques and type of surgery (elective vs emergency) were recorded in just 4 (14.2%). Operative diagnosis and time of surgery were each present in 8 (28.5%) notes. Incision details and findings were mentioned in 7 (25.0%), while documentation of complications was rare. Only 5 (17.8%) notes were signed, and DVT prophylaxis was not mentioned at all.

In the second cycle, conducted after the intervention, significant improvements were observed. Closure techniques were documented in 25 (75.8%) notes, compared to 4 (14.2%) pre-intervention. The surgeon’s name, operative diagnosis, and postoperative instructions were recorded in 25 (75.8%), while the anesthetist’s name and operation time were present in 22 (66.7%). The date of operation was included in 24 (72.7%), the assistant’s name in 23 (69.7%), and the type of surgery in 18 (54.5%). Documentation of operative findings improved to 21 (63.6%), and incision details to 19 (57.6%). Details of tissue removed or added were included in 14 (42.4%), and anticipated blood loss in the same percentage. DVT prophylaxis was documented in 8 (24.2%), and signatures rose to 13 (39.4%). Table 1 illustrates the most notable improvements, observed in closure technique (+61.6%), surgeon’s name (+43.7%), and operative diagnosis (+47.3%). Interestingly, postoperative instructions declined from 92.8% to 75.8%. This may reflect reliance on pre-existing routine ward round documentation for postoperative care, rather than duplication in the operative note, especially under staff and time pressures. Table 2 illustrates the compliance of documentation across both audit cycles.

**Table 1:** Comparison of documentation parameters between the two audit cycles. DVT: Deep vein thrombosis.

Parameter	First cycle N (%)	Second cycle N (%)	Improvement (%)
Postoperative instructions	26 (92.8%)	25 (75.8%)	-17.00%
Date of operation	9 (32.1%)	24 (72.7%)	0.406
Operating surgeon’s name	9 (32.1%)	25 (75.8%)	0.437
Assistant’s name	9 (32.1%)	23 (69.7%)	0.376
Anaesthetist’s name*	9 (32.1%)	22 (66.7%)	0.346
Operative diagnosis	8 (28.5%)	25 (75.8%)	0.473
Time of operation	8 (28.5%)	22 (66.7%)	0.382
Type of surgery	4 (14.2%)	18 (54.5%)	0.403
Closure technique	4 (14.2%)	25 (75.8%)	0.616
Complications	4 (14.2%)	10 (30.3%)	0.151
Operative findings	7 (25.0%)	21 (63.6%)	0.386
Incision details	7 (25.0%)	19 (57.6%)	0.326
Tissue removed/added	1 (3.5%)	14 (42.4%)	0.389
Prosthesis details	1 (3.5%)	4 (12.1%)	0.086
Antibiotic prophylaxis	1 (3.5%)	10 (30.3%)	0.268
Anticipated blood loss	1 (3.5%)	14 (42.4%)	0.389
DVT prophylaxis	0 (0.0%)	8 (24.2%)	0.242
Signature	5 (17.8%)	13 (39.4%)	0.216
Additional procedures	2 (7.1%)	2 (6.1%)	-1.00%

**Table 2:** Mean compliance comparison across audit cycles.

Audit cycle	Mean compliance (%)
First cycle	24.10%
Second cycle	52.30%
Improvement	0.282

These improvements highlight the effectiveness of structured templates and targeted training in enhancing operative note quality.

## Discussion

This audit demonstrates significant improvements in operative note documentation following the introduction of a standardized template and brief training sessions. Key parameters that were previously poorly documented, such as closure methods, operating surgeon, anesthetist, and operative findings, showed substantial improvement. The wartime context is central to our findings: even under disrupted staffing and resource shortages, simple structured tools produced measurable gains, underscoring their adaptability to unstable environments.

Our findings are consistent with previous Sudanese audits, which have shown that structured proformas and staff education enhance the completeness of operative notes across multiple centers, including Dongola, Port Sudan, Elobeid, Doka, Wad Madani, and Atbara [1-6]. International studies also confirm similar trends, with audits from Pakistan, Malawi, and the UK reporting improved compliance following the introduction of proformas and training [7-11].

By conducting a closed-loop audit, we were able to assess and improve surgical operative note documentation. Using a standardized checklist aligned with RCSEng guidelines, the team implemented structured templates and staff education. Post-intervention results showed dramatic improvements: patient identification rose from 2% to 100%, preoperative diagnosis from 8% to 93.5%, and postoperative care instructions from 0% to 76.1% (all p < 0.0001). Despite persistent gaps in complication reporting and anesthetist details, the study underscores the effectiveness of low-cost, scalable interventions in enhancing documentation quality, even amid conflict-related disruptions. It adds valuable insight into the adaptability of audit-driven improvements in resource-limited and crisis-affected settings.

Despite overall improvement, the magnitude of change in our audit (+28.2%) was smaller compared to some peacetime hospitals, where compliance increased by over 40% [12]. Our audit achieved an increase from 24.1% to 52.3% (+28.2%). While the magnitude of improvement was smaller in our setting, this is likely attributable to the lower baseline compliance, wartime staff shortages, and broader case mix beyond a single procedure type. The UK audit also found large gains in key fields after redesigning operative notes, underscoring the universal benefit of structured formats [13]. A ward round documentation study showed that certain fields remain difficult to consistently complete, similar to our findings with postoperative instructions and DVT prophylaxis [14]. Finally, an electronic proforma audit improved compliance from ~80% to >95%, with emphasis on training junior staff, relevant as most of our notes were authored by trainees [15].

This audit contributes further evidence from a wartime context, underlining the adaptability and impact of low-cost quality improvement interventions in resource-constrained settings. The minor decline in postoperative instructions might reflect variability in adherence, due to shifting responsibility to ward round notes, a perception that instructions were already verbally communicated, or reduced compliance caused by staff fatigue during conflict. These issues could be addressed through continuous monitoring.

Limitations

This audit had several limitations. It was conducted during an active conflict in Sudan, which led to staff shortages, high turnover, and disruptions in hospital services that may have affected both documentation practices and intervention implementation. There was also potential for observer bias, as data abstraction may have been influenced post-intervention. The relatively small sample size and single-center design limit the generalizability of findings to other settings. Furthermore, the study focused on quantitative assessment of compliance with RCSEng guidelines and did not explore qualitative factors such as clinicians’ attitudes, workload pressures, or barriers to proper documentation. The short post-intervention period also precluded evaluation of the long-term sustainability of improvements. Finally, reliance on paper-based records in a resource-limited environment posed challenges in achieving uniform compliance despite the introduction of templates and reminders.

## Conclusions

The implementation of structured documentation and brief training at Abuhamad Hospital led to marked improvements in the completeness and clarity of operative notes, demonstrating the efficacy of targeted, low-cost interventions in enhancing clinical record-keeping. These results highlight the critical role of standardized templates in promoting consistency and reducing variability in documentation practices, particularly in settings where resources and personnel may be constrained. Moreover, the success of brief educational sessions underscores the importance of ongoing professional development, even in high-turnover or conflict-affected environments. To sustain these gains, routine auditing should be institutionalized as part of broader quality assurance frameworks, enabling timely identification of gaps and reinforcing adherence to best practices. Ultimately, this audit reinforces the principle that even modest interventions, when strategically implemented, can yield substantial improvements in patient safety, medicolegal protection, and clinical communication. In the long term, transitioning from paper-based templates to digital or electronic documentation systems, where feasible, could further enhance sustainability, legibility, and accessibility of operative records.
